# S-DCNN: prediction of ATP binding residues by deep convolutional neural network based on SMOTE

**DOI:** 10.3389/fgene.2024.1513201

**Published:** 2025-01-06

**Authors:** Sixi Hao, Cai-Yan Li, Xiuzhen Hu, Zhenxing Feng, Gaimei Zhang, Caiyun Yang, Huimin Hu

**Affiliations:** ^1^ College of Sciences, Inner Mongolia University of Technology, Hohhot, China; ^2^ School of Mathematics and Statistics, Xinyang College, Xinyang, China; ^3^ School of Computer Science and Technology/Baotou Medical College, Baotou, China; ^4^ Department of Obstetrics and Gynecology, Hohhot First Hospital, Hohhot, China

**Keywords:** ATP binding residues, synthetic minority over-sampling technique, deep convolutional neural network, propensity factors, dihedral angle, energy

## Abstract

**Background:**

The realization of many protein functions requires binding with ligands. As a significant protein-binding ligand, ATP plays a crucial role in various biological processes. Currently, the precise prediction of ATP binding residues remains challenging.

**Methods:**

Based on the sequence information, this paper introduces a method called S-DCNN for predicting ATP binding residues, utilizing a deep convolutional neural network (DCNN) enhanced with the synthetic minority over-sampling technique (SMOTE).

**Results:**

The incorporation of additional feature parameters such as dihedral angles, energy, and propensity factors into the standard parameter set resulted in a significant enhancement in prediction accuracy on the ATP-289 dataset. The S-DCNN achieved the highest Matthews correlation coefficient value of 0.5031 and an accuracy rate of 97.06% on an independent test set. Furthermore, when applied to the ATP-221 and ATP-388 datasets for validation, the S-DCNN outperformed existing methods on ATP-221 and performed comparably to other methods on ATP-388 during independent testing.

**Conclusion:**

Our experimental results underscore the efficacy of the S-DCNN in accurately predicting ATP binding residues, establishing it as a potent tool in the prediction of ATP binding residues.

## Introduction

Adenosine 5′-triphosphate (ATP) is an unstable high-energy phosphate compound. It interconverts with adenosine 5′-diphosphate (ADP) to achieve energy storage and release in cells and ensures the energy supply of various life activities of cells. As an important ligand, ATP also plays a critical role in the realization of protein functions (Chauhan et al., 2009). For example, ATP binds with myosin to provide energy and promotes its combination with actin to form cross bridges, which is used to regulate muscle contraction (Holmes et al., 2003); the combination of ATP and sodium-potassium ATPase can regulate the concentration of intracellular sodium/potassium ions, thus maintaining the resting potential of the cell (Kanai et al., 2013). In fact, the protein-ATP interactions depend on ATP binding residues on proteins. Therefore, accurate prediction of ATP binding residues is of great value for understanding protein function, disease occurrence and molecular drug design.

At present, substantial advancements have been achieved in the prediction of ATP binding residues from protein sequences (Guo et al., 2005; Chauhan et al., 2009; Song et al., 2020b). In 2017, Ding et al. (2017) utilized the ATP-221 dataset devised by Yu et al. (2013), extracting features from the discrete cosine transform of the position-specific scoring matrix (PSSM) and predicted relative solvent accessibility. They employed the random under-sampling (RUS) technique and weighted sparse representation-based classifier (WSRC) to predict protein-ATP binding sites, achieving a Matthews correlation coefficient (MCC) of 0.506 and an accuracy (ACC) of 96.8% on an independent test set. Similarly, Zhao et al. (2019) introduced an SXGBsite prediction model in the same year, utilizing PSSM and predicted relative solvent accessibility as parameters with the extreme gradient boosting algorithm. The prediction performance yielded MCC and ACC values of 0.463% and 96.5%, respectively, on the independent test set. Nguyen et al. (2019) developed a tool of DeepATP for predicting ATP-binding sites in membrane proteins, which combined evolutionary information in the form of PSSM and two-dimensional convolutional neural network. In 2020, our research group (Hu et al., 2020) constructed a new dataset, ATP-289, and selected amino acids, hydrophilic-hydrophobic, polarity, predicted secondary structure, and relative solvent accessibility as feature parameters. By utilizing random undersampling with the support vector machine (SVM) algorithm, the MCC value reached 0.549 with 5-fold cross-validation. Additionally, Song et al. (2020a) utilized the ATP-388 dataset, choosing PSSM, predicted secondary structure, predicted relative solvent accessibility, and one-hot encoding as feature parameters. They applied class-weighted ensemble deep learning algorithms, achieving ACC and MCC values of 97.2% and 0.626, respectively, on the independent test set. In 2021, Hu et al. (2021) introduced the novel method DeepATPseq, achieved ACC and MCC values of 57.42% and 0.655, respectively, on the independent ATP-388 test set. Nguyen et al. (2022) applied multiple convolutional window scanning filters of a convolutional neural network on PSSM to predict ATP-binding sites, and the resulting model outperformed other algorithms on the same datasets.

In summary, previous studies have primarily enhanced the prediction accuracy of ATP binding residues in three main areas. Firstly, sampling techniques were frequently applied to address the significant imbalance between positive and negative samples. Secondly, novel feature parameters and extraction methods were integrated into the prediction models. Lastly, a variety of traditional machine learning algorithms and deep learning methods were utilized for prediction tasks.

This study introduced the S-DCNN method to enhance the accuracy of predicting ATP binding residues. A balanced dataset was created using the SMOTE algorithm, which preserved information integrity. New parameters, including dihedral angles, energy, and propensity factors, were introduced. Furthermore, the DCNN algorithm with three optimized hyperparameters enhanced the prediction of ATP binding residues. Finally, the S-DCNN was applied to two additional datasets to validate the model.

## Materials and methods

### Datasets

The ATP dataset utilized in this study was constructed by our group (Hu et al., 2020) through the following steps: initially, 1728 ATP protein chains were sourced from the semi-manual BioLip database (Yang et al., 2013); subsequently, these chains were filtered with sequence length (>50 residues), the resolution (<3 Å), and the sequence identity (<30%); resulting in the ATP-289 dataset with 289 protein chains including 3901 ATP binding residues and 104153 ATP non-binding residues; the dataset was then partitioned randomly into training and testing sets, with the former containing 260 protein chains encompassing 3526 ATP binding residues and 92804 non-binding residues, and the latter comprising 29 protein chains with 375 ATP binding residues and 11349 ATP non-binding residues. We performed five-fold cross-validation using the training set to obtain the trained model and validated the model’s effectiveness using an independent test set. The source codes and datasets in this study are available at https://github.com/tlhsx/S-DCNN.

Previous researches indicated that residues neighboring binding sites can influence ligand interactions with these sites (Hu et al., 2016; Liu et al., 2019). To address this, protein sequences were segmented into fragments using the sliding window method, ensuring each amino acid resided at the fragment center by adding (L-1)/2 pseudo-amino acids at both sequence ends. Here, L denotes the fragment length. If a residue of (L + 1)/2 was the binding residue, it was defined as a positive sample, otherwise, it was a negative sample. Based on the previous references (Yu et al., 2013; Ding et al., 2017; Zhao et al., 2019; Hu et al., 2020; Hu et al., 2021), the intercepted fragment L was 17.

### Statistical analysis and reclassification of predicted dihedral angle

**FIGURE 1:**
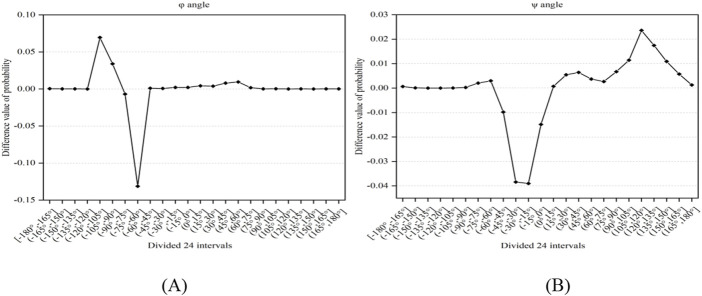
The difference value of probability between positive and negative samples. Note: **(A, B)** represent the φ and ψ angles, respectively. The *x*-axis represents the divided 24 intervals, the *y*-axis represents the difference value of probability between positive and negative samples.

The secondary structure of proteins can reflect the trend of the backbone chain, and the dihedral angle is the main descriptor of the secondary structure, which can reflect the local structural information of proteins and is a very effective feature for predicting protein-ligand binding residues (Chen et al., 2011; Cui et al., 2019; Liu et al., 2020). Here, we applied firstly the reclassified dihedral angles to the prediction of ATP binding residues. First, the values of phi (φ) and psi (ψ) angles were obtained from the primary sequence using ANGLOR software (Wu and Zhang, 2008), and the value range of φ and ψ angles both were [−180°, 180°]; then every 15° was divided into an interval, the φ and ψ angles both were divided into 24 intervals; the difference value of probability of the φ and ψ angles between the positive and negative samples was obtained, as shown in Figures 1A, B. The formula for calculating the probability difference is expressed in Equation 1 as follows:

$$\Delta P_{i}=P_{i+}-P_{i-}$$ (1)

where, $P_{ij}=\frac{n_{ij}}{\sum_{i=1}^{24}n_{ij}},$ *n*_*ij*_ represents the number of *i* ^th^ interval in positive or negative samples; *i* (*i* = 1, 2, … , 24) represents the divided interval; *j* (+ or -) represents a positive or negative sample.

In Figure 1, it was found that there exist significant differences in the probabilities of the φ and ψ angles between the positive and negative samples. Using 0 as the threshold, we divided the φ and ψ angles into three intervals, which were represented by functions g(x) and h(x) (i.e., Equations 2, 3). Through the above analysis, we selected the reclassification information of the φ and ψ angles as features.

$$g\left(x\right)=\left\{\begin{array}{l} I,x\in \left[-180^{\circ },-90^{\circ }\right]\\ \mathrm{II},x\in \left(-90^{\circ },-60^{\circ }\right]\\ \mathrm{III},x\in \left(-60^{\circ },180^{\circ }\right] \end{array}\right.$$ (2)

$$h\left(x\right)=\left\{\begin{array}{l} I,x\in \left[-180^{\circ },-60^{\circ }\right]\\ \mathrm{II},x\in \left(-60^{\circ },0^{\circ }\right]\\ \mathrm{III},x\in \left(0^{\circ },180^{\circ }\right] \end{array}\right.$$ (3)

### Statistical analysis and reclassification of energy values

**FIGURE 2:**
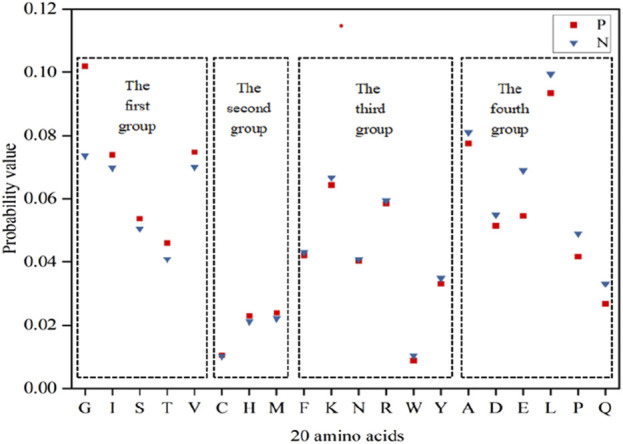
Energy probability of 20 amino acids in positive and negative samples. Note:The *x*-axis represents 20 amino acids, the *y*-axis represents the probability value, and P and N represent the positive and negative samples, respectively.

In accordance with the principles of physics, the stability of molecular structures increases as energy decreases (Wang et al., 2021). In consideration of the specificity of ATP binding to proteins, we analyzed the Laplace energy values of the 20 amino acids between positive and negative samples in Figure 2. The analysis revealed varying energy probabilities among the 20 amino acids between the positive and negative samples. Consequently, the amino acids were regrouped into four categories: the first group comprised G, I, S, T and V, with markedly higher values in the positive set than in the negative set; the second group included C, H and M, where the positive set’s values slightly surpassed those of the negative set; the third group encompassed F, K, N, R, W and Y, in which the values of negative set were slightly higher than that of positive set; the fourth group consisted of A, D, E, L, P and Q, with notably higher values in the negative set than in the positive set. Subsequently, the energy reclassification details were utilized as feature parameters for ATP binding residue identification.

### Propensity factors feature

**FIGURE 3:**
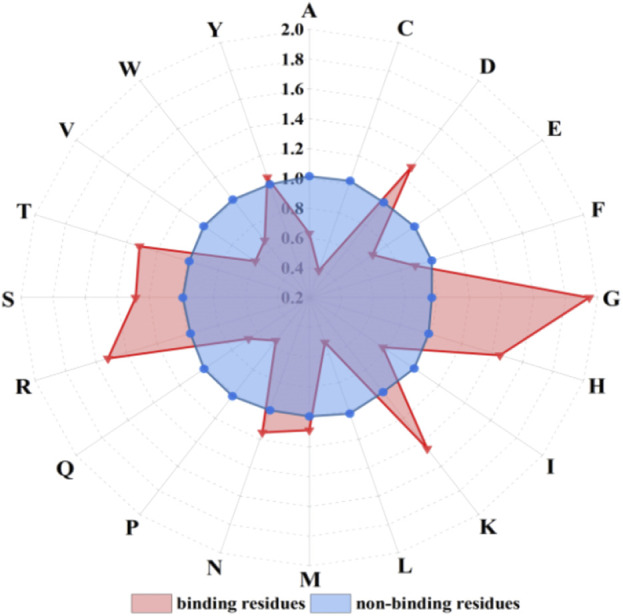
The propensity factors of 20 amino acids of binding residues and non-binding residues. Note: letters on the radius represent 20 amino acids; red triangles and blue dots represent amino acid propensity factor values of binding residues and non-binding residues, respectively.

Researchers have analyzed the influence of binding residues and their neighboring residues on the protein-ATP binding process at the sequence fragment level. In protein-ligand interactions, the amino acids’ specific preferences in crucial binding residues that directly engage with the ligand play a vital role in the binding process. Hence, we introduced a novel parameter extraction method termed propensity factors. Originally suggested by Chou and Fasman (1974), propensity factors have found utility in predicting protein secondary structures and ion ligand-binding sites (Chou and Fasman, 1979; Xu et al., 2022). The formula was expressed in Equation 4 as follows:

$$F_{ij}=\frac{p_{ij}}{p_{j}}$$ (4)

where, $p_{ij}=\frac{n_{ij}}{N_{i}}$ , $p_{j}=\frac{N_{j}}{N_{t}}$ , $N_{i}=\sum_{i=1}^{20}n_{ij}$ , $N_{t}=\sum_{j=1}^{2}N_{j}$ , *n*_*ij*_ represents the number of amino acid *i* in binding residues or non-binding residues; *N*_*j*_ represents the number of binding residues or non-binding residues; *i* (*i* = 1, 2, … , 20) represents 20 amino acids; *j* (*j* = 1, 2) represents binding residues and non-binding residues. The propensity factors of 20 amino acids were statistically analyzed, as shown in Figure 3.

The propensity factor values of amino acids D, G, H, K, R, S, and T within the binding residues exhibited notably higher values compared to those in the non-binding residues. Hence, the propensity factors, serving as a novel extraction method, effectively capture the preferences of the binding residues.

### Basic features

Utilizing sequence information, we extracted amino acids and derived predicted secondary structure information, relative solvent accessibility, and the hydrophilic-hydrophobic profile as fundamental features. These parameters, extensively employed in prior research, have demonstrated exceptional predictive capabilities (Chen et al., 2011; Zhang et al., 2012; Yu et al., 2013; Hu et al., 2016; Ding et al., 2017; Zhao et al., 2019; Hu et al., 2020; Song et al., 2020a; Hu et al., 2021). The hydrophilic-hydrophobic properties were used to classify the 20 amino acids into six distinct categories (Pánek et al., 2005). Secondary structure and solvent accessibility predictions were generated through ANGLOR software, categorizing secondary structure into α-helix, β-sheet and coil. Following guidelines from a source (Hu et al., 2020), relative solvent accessibility predictions were partitioned into four intervals: (0, 0.2], (0.2, 0.45], (0.45, 0.6], (0.6, 0.85].

### Composition and site conservation information

The researchers observed significant disparities in amino acid frequencies between positive and negative samples, prompting the utilization of amino acid composition as a parameter (Hu et al., 2020; Wang et al., 2021; Sun et al., 2022). Here, from the amino acids composition, secondary structure, relative solvent accessibility, φ angle, ψ angle and energy, we extracted 21, 4, 5, 4, 4 and 5-dimensional composition information, respectively.

Previous studies have shown that the position weight matrix can well reflect the site conservation of amino acids in protein sequences (Hu et al., 2020; Xu et al., 2022). Here, the matrix elements were expressed in Equation 5 as follows:

$$m_{i,j}=\ln\left(\frac{p_{i,j}}{p_{0,j}}\right)$$ (5)

where, $p_{i,j}=\frac{\left(n_{i,j}+\frac{\sqrt{N_{i}}}{q}\right)}{\left(N_{i}+\sqrt{N_{i}}\right)}$ , $N_{i}=\sum_{j=1}^{21}n_{i,j}$ , *P*_*0,j*_ represents the background probability, and *n*_*i,j*_ represents the frequency of the *j* ^th^ amino acid at the *i* ^th^ site, *j* represents 20 kinds of amino acids and vacancies, *q* represents the number of classifications, here it is 21. Two standard scoring matrices were obtained from the positive and negative training sets, and 2L-dimensional feature vector were obtained for each segment. Similarly, the predicted secondary structure (*q* = 4), relative solvent accessibility (*q* = 5), energy (*q* = 5), φ angle (*q* = 4) and ψ angle (*q* = 4) were also extracted by the same methods, and a total of 6 × 2L-dimensional site conservative information was obtained.

### Information entropy

The intermolecular hydrophobic effect was a complex process which was mainly determined by the entropy effect (Wang et al., 2021). The information entropy was an effective method to extract information of hydrophilic-hydrophobic (Wang et al., 2021; Sun et al., 2022; Xu et al., 2022). Here, the information entropy formula was expressed in Equation 6 as follows:

$$H\left(x\right)=-\sum_{j=1}^{q}p_{j}\log_{2}p_{j}$$ (6)

where, $p_{j}=\frac{n_{j}}{N}$ , *n*_*j*_ represents the frequency of occurrence of the *j* ^th^ classification in a segment, *N* is the segment length, and *q* represents the hydrophilic-hydrophobic classifications and vacancies, here it is *q* = 7.

### Algorithm

#### SMOTE algorithm

The number of non-binding residues was far greater than the that of binding residues, and the serious imbalance of data would lead to a high false positive. For this reason, researchers often used random undersampling technology to process the dataset, which randomly selected the same number as the binding residue sample from the non-binding residue sample to construct a balanced set. The disadvantage of this method was the loss of non-binding residue sample information. To overcome the above limitations, we employed an over-sampling technique: SMOTE (Chawla et al., 2002). It generated the same number of non-binding residue samples from binding residue samples to construct a balanced training set. For each sample of the binding residue, the SMOTE algorithm calculated the distance (i.e., Euclidean distance) between the point and other binding residue sample points and selected the nearest k binding residue samples; then a sample point was randomly selected from the k sample points, the two points drew a line segment; finally it generated a new sample point by interpolation operation on the line segment, where k was the default value. This technique ensured that sample information would not be lost and the data had integrity. The SMOTE algorithm was different from the random over-sampling technique, and the newly generated sample was obtained by the analysis of the binding residue sample rather than direct copy, so it not only conformed to the generality of the binding residue sample, but also differed from each binding residue sample, which can effectively solve the classification over-fitting problem caused by the small decision interval. In the sample space, SMOTE generated new samples according to the following Equation 7:

$$x_{new}=x_{old}+rand\left(0,1\right)\times \left(x^{\prime }-x_{old}\right)$$ (7)

where, $x_{old}$ represents the ATP binding residue sample in the training set, $x_{new}$ represents the newly generated ATP binding residue sample, and $x^{\prime }$ represents a sample of ATP binding residue randomly selected from k neighbors of $x_{old}$ .

### Deep convolutional neural network (DCNN)

As one of the most important branches of deep learning framework, DCNN usually consisted of the input layer, convolutional layer, pooling layer, fully connected layer and output layer. The potential complex information was detected for the input raw data, and then through a series of high-dimensional and high-level projection mapping, the deeper representation information of the classified objects was obtained. The alternating distribution of the convolutional layer and the pooling layer made the convolutional neural network had better fault tolerance and parallel processing ability, and the generalization ability and adaptability of the model were greatly enhanced. It has been widely used in various fields.

The DCNN model framework in this paper was implemented by Keras, and the bottom layer was based on the TensorFlow framework. Here, the batch normalization was employed to avoid vanishing gradients and speed up the convergence of the network. In order to prevent over-fitting of the model, the layer of dropout was employed. The relu nonlinear activation function was used to improve the expressive ability of the model and greatly shortened the learning cycle. To effectively avoid over-fitting problems caused by continued training, the early stopping module was used. Adam and cross-entropy were used as optimizer and loss function, respectively. The output layer applied the sigmoid function to make the classification objects output probability values between 0 and 1. The hyperparameters in DCNN algorithm had an influence on the training speed and performance of the predictor. Based on the previous research, we mainly optimized the following three hyperparameters. Here, the dropout was set as 0.2; the range of the number of convolutional layers was from 1 to 6; the range of filters and batch size was both from 2 to 128. Detailed description of DCNN architecture can be viewed at https://github.com/tlhsx/S-DCNN.

### Validation methods and evaluation metrics

**FIGURE 4:**
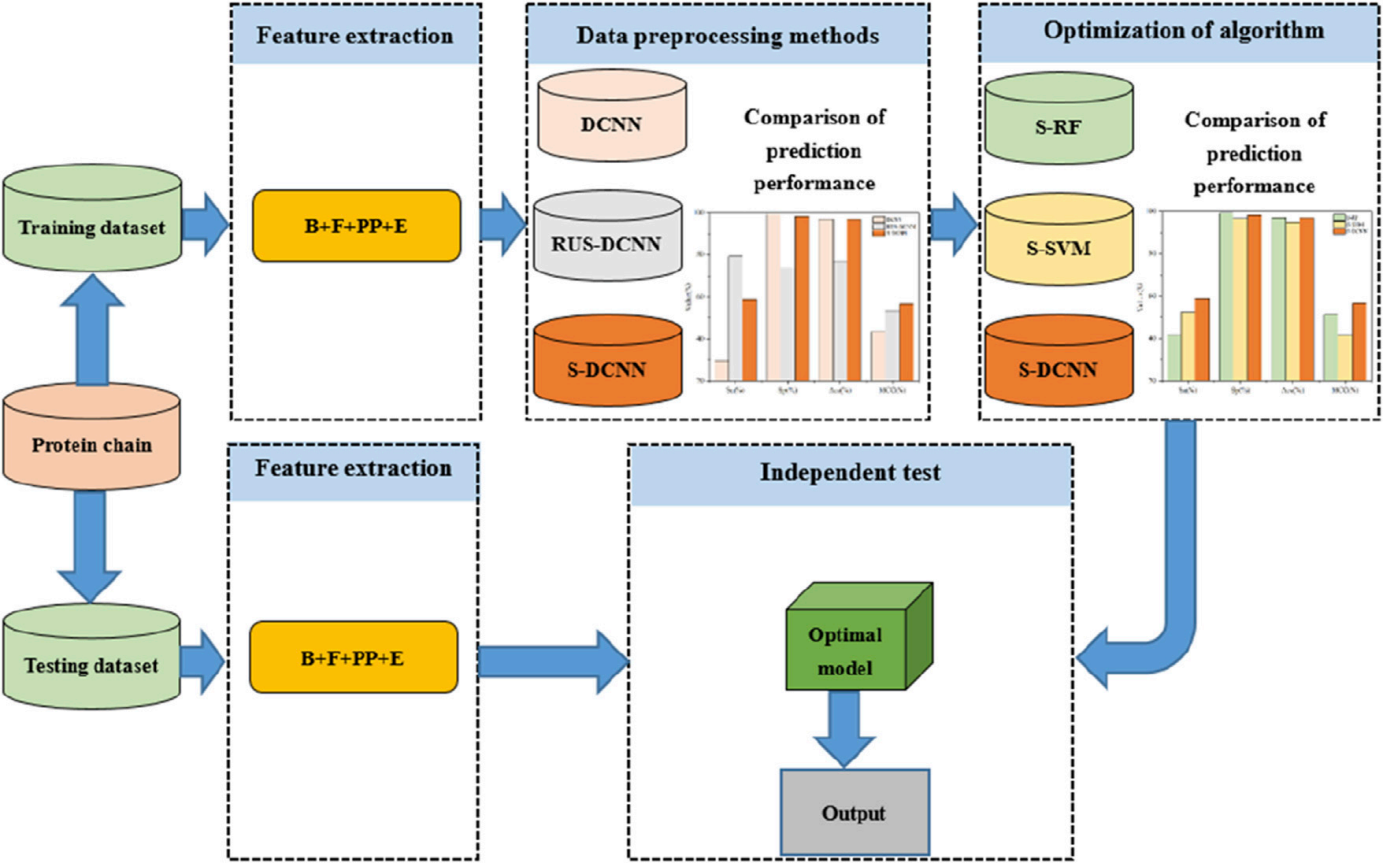
Flowchart of the proposed method for predicting ATP binding residues. Note: B, F, PP, and E represent basic features, propensity factors, dihedral angle, and energy, respectively; DCNN, RUS-DCNN, and S-DCNN represent DCNN predictors with using samples without preprocessing, random undersampling, and SMOTE, respectively; S-RF and S-SVM stand for RF and SVM predictors based on SMOTE, respectively.

The validation methods in this paper were 5-fold cross-validation and independent testing. For the evaluation of the prediction results, we adopted the evaluation indicators commonly used in the identification of ATP binding residues: sensitivity (*S*_*n*_), specificity (*S*_*p*_), accuracy (*ACC*), and Matthews correlation coefficient (*MCC*) (i.e., Equations 8–11) (Zou et al., 2023).

$$S_{n}=\frac{TP}{TP+FN}\times 100\%$$ (8)

$$S_{P}=\frac{TN}{TN+FP}\times 100\%$$ (9)

$$A\text{CC}=\frac{TP+TN}{TP+TN+FP+FN}\times 100\%$$ (10)

$$MCC=\frac{\left(TP\times TN\right)-\left(FP\times FN\right)}{\sqrt{\left(TP+FP\right)\left(TP+FN\right)\left(TN+FP\right)\left(TN+FN\right)}}$$ (11)

where, the number of ATP binding residues correctly predicted is *TP*, otherwise it is *FN*; the number of ATP non-binding residues correctly predicted is *TN*, otherwise it is *FP*. In addition, the flowchart was clearly described in Figure 4.

## Results

### Prediction results of basic feature parameters

The basic feature parameters were input into the S-DCNN predictor, and the results of the 5-fold cross-validation were shown in Table 1. Here, the S_n_ and MCC values were 43.39% and 0.4101, respectively.

**TABLE 1:** The prediction results of 5-fold cross-validation.

Features	S_n_(%)	S_p_(%)	ACC(%)	MCC	Hyperparameter
B	43.39	97.82	95.83	0.4101	4,16,16
B+E	44.36	97.83	95.90	0.4176	4,16,16
B+PP	46.15	97.91	96.04	0.4363	4,16,16
B+F	47.82	97.93	96.12	0.4509	4,16,16
B+F+E	48.47	97.96	96.15	0.4597	4,16,16
B+F+PP	49.32	97.96	96.18	0.4663	4,16,16
B+F+PP+E	50.90	98.01	96.31	0.4773	4,16,16
(B+F+PP+E)*	58.82	98.40	96.97	0.5681	3,16,32

Note: B, F, PP, and E represent basic features, propensity factors, dihedral angle and energy, respectively; () * represents the prediction results after optimization of hyperparameters; the three hyperparameters are the number of convolution layers, filters and batch size, respectively.

### Prediction results of adding dihedral angle, energy and propensity factors

To improve the prediction performance, the dihedral angle, energy and propensity factors were introduced. The extracted dihedral angle, energy, propensity factor feature parameters and basic feature parameters were fused and input to the S-DCNN predictor, and the results were shown in Table 1 on 5-fold cross-validation.

In Table 1, when the feature parameters PP, E or F were respectively added to the feature B, the prediction results of B + F were relatively better. Then, the parameter PP or E was added to the above parameter set of B + F, and the results with parameter set of B + F + PP were relatively better. When the feature parameters PP, E and F were added at the same time, the best prediction results were obtained. The S_n_, S_p_, ACC and MCC values with feature set of B + F + PP + E reached 50.9%, 98.01%, 96.31% and 0.4773, respectively.

### Optimization of hyperparameters

**FIGURE 5:**
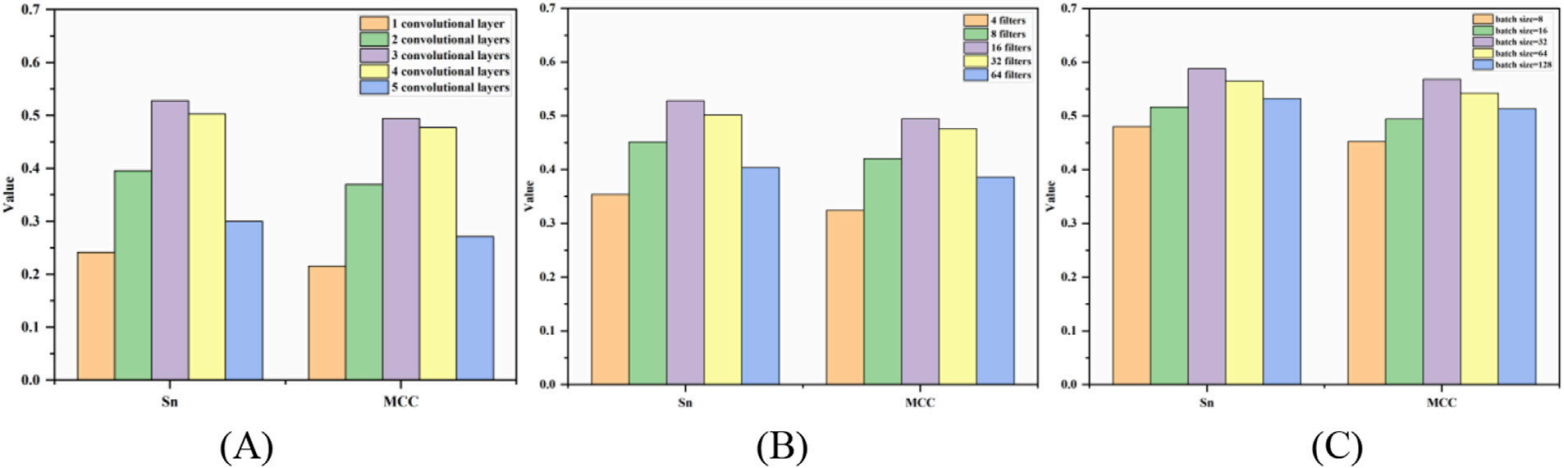
Bar chart of S_n_ and MCC values changing with hyperparameters. Note: **(A–C)** represent the optimization of the number of convolution layers, filters and batch size, respectively, and the *y*-axis is the value of MCC and S_n_.

The prediction results of the three hyperparameters of the 5-fold cross-validation were shown in Figure 5. Figure 5A was a bar chart of the MCC and S_n_ values changing with the number of convolution layers. When the number of layers was 3, the S_n_ and MCC values reached the peak at the same time, then the optimal number of layers is 3. From Figures 5B, C, the optimal filters and batch size were 16 and 32, respectively.

The prediction results after optimization of hyperparameter were shown in (B + F + PP + E)* of Table 1. The S_n_, S_p_, ACC and MCC values reached 58.82%, 98.4%, 96.97%, and 0.5681, respectively.

### Prediction results of DCNN algorithm with different preprocessing methods

To assess the efficacy of the SMOTE algorithm in predicting ATP binding residues, we conducted a comparative analysis between SMOTE and random undersampling alongside samples without preprocessing (referred to as RUS-DCNN and DCNN, respectively). In RUS-DCNN, for result stability, negative set samples were randomly selected ten times, with the average outcome of these ten selections serving as the final prediction. The prediction results of 5-fold cross-validation after optimization of hyperparameter were listed in Table 2. In Table 2, the MCC values of RUS-DCNN, DCNN and S-DCNN reached more than 0.438, and the ACC values of DCNN and S-DCNN reached more than 96.97%.

**TABLE 2:** Comparison of prediction results.

Methods	S_n_(%)	S_p_(%)	ACC(%)	MCC	Hyperparameter
DCNN	29.75	99.49	96.97	0.4385	3,32,32
	−29.07	+1.09	0	−0.1296	
RUS-DCNN	79.26	74.24	76.75	0.5357	2,32,32
	+20.44	−24.16	−20.22	−0.0324	
S-DCNN	58.82 (49.20)	98.40 (98.64)	96.97 (97.06)	0.5681 (0.5031)	3,16,32
S-SVM	52.69 (44.27)	96.65 (97.95)	95.06 (96.23)	0.4171 (0.4097)	—
	−6.13 (−4.93)	−1.75 (−0.69)	−1.91 (-0.83)	−0.151 (−0.0934)	
S-RF	41.76 (44.53)	99.21 (98.94)	97.10 (97.20)	0.5140 (0.4950)	—
	−17.06 (−4.67)	+0.81 (+0.3)	+0.13 (+0.14)	−0.0541 (−0.0081)	

Note: values in brackets are the prediction results of independent testing; DCNN, RUS-DCNN, and S-DCNN, represent DCNN, predictors with using raw dataset without preprocessing, random undersampling, and SMOTE, respectively; S-RF, and S-SVM, stand for RF, and SVM, predictors based on SMOTE, respectively; the second row of each method represents the difference between the results of the method and the S-DCNN method, “+” and “−” represent the increase and decrease of the prediction performance over the S-DCNN method, respectively; the three hyperparameters are the number of convolution layers, filters and batch size, respectively.

### Prediction results of different algorithms based on SMOTE

To verify the superiority of the S-DCNN algorithm, we computed the results of the SVM and random forest (RF) algorithm with SMOTE(i.e., S-SVM and S-RF) through 5-fold cross-validation, as detailed in Table 2. Specifically, the RF model utilized 500 decision trees, the SVM model employed a radial basis function kernel, and other parameters remained at default values.

**FIGURE 6:**
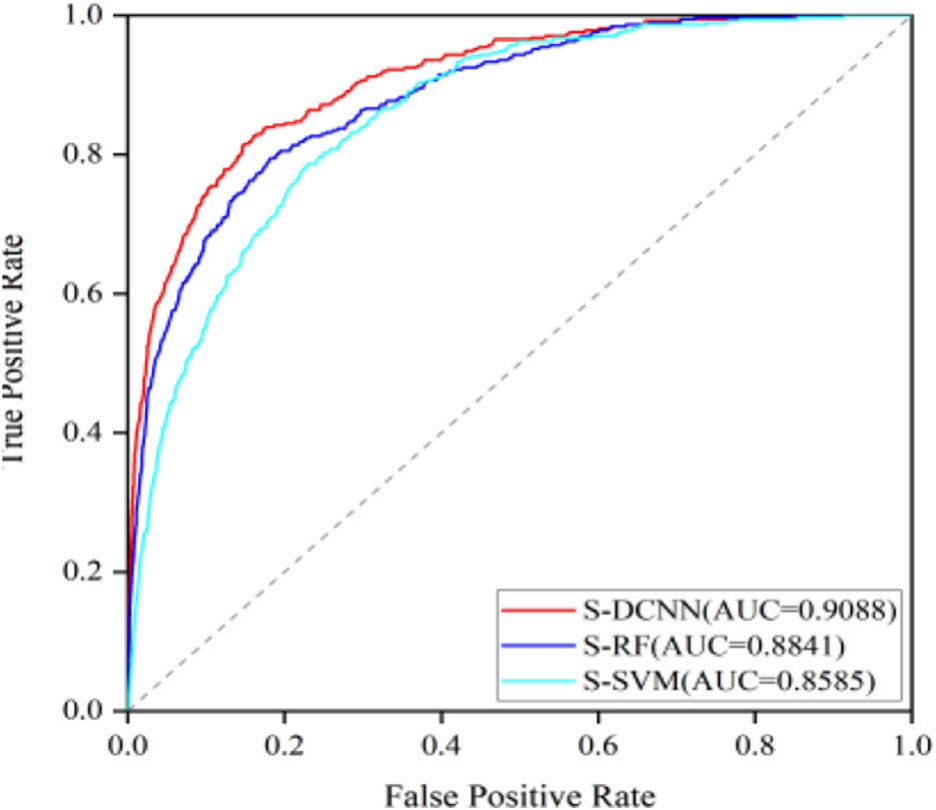
ROC curves of different algorithms based on SMOTE on ATP-289 independent testing set.

To test the generalization ability of the prediction model, an independent testing set was utilized to predict ATP binding residues with the corresponding results outlined in Table 2 within brackets. Across the evaluation metrics of MCC and ACC, S-SVM, S-RF, and S-DCNN achieved values exceeding 0.409% and 96.2% respectively. Notably, S-DCNN demonstrated superior performance in terms of S_n_ and MCC. Moreover, the prediction model’s performance was assessed using the area under the Receiver Operating Characteristic (ROC) curve (AUC). Figure 6 illustrates the ROC curves for various algorithms based on SMOTE on the ATP-289 independent testing set, where S-SVM, S-RF, and S-DCNN yielded AUC values of 0.8585, 0.8841, and 0.9088 respectively.

### Comparison with previous results

To further verify the prediction performance of S-DCNN, we applied S-DCNN on another two frequently used datasets. The first set was constructed by Yu et al. (2013), in which the training set had 221 protein chains (ATP-221), and the independent testing set had 50 protein chains (ATP-50). The other set was constructed by Hu et al. (2018), in which the training set had 388 protein chains (ATP-388), and the independent testing set had 41 protein chains (ATP-41).

### Prediction results on the 5-fold cross-validation

Using 5-fold cross-validation with optimized hyperparameters, the S-DCCN method was performed on the ATP-221 and ATP-388 datasets. The corresponding two prediction results were shown in Tables 3, 4, respectively. In Table 3, the S-DCNN achieved ACC of 97.0% on the ATP-221 dataset, surpassing other methods by 0.6%–0.8%. The MCC of the S-DCNN also exhibited significant improvement, with an increase ranging from 3.6% to 12.5%. In Table 4, the ACC and MCC values of the S-DCNN on the ATP-388 dataset reached 97.04% and 0.5887, respectively. To make a better comparison, we also listed the prediction results of the previous on the ATP-221 and ATP-388 datasets.

**TABLE 3:** Comparison of prediction performance on ATP-221 dataset.

Method	S_n_(%)	S_p_(%)	ACC(%)	MCC	Hyperparameter
S-DCNN	58.4 (50.2)	98.5 (98.8)	97.0 (97.0)	0.573 (0.545)	3,32,32
SXGBsite	40.3 (43.7)	98.6 (98.5)	96.4 (96.5)	0.448 (0.463)	−
EC-RUS	58.6 (45.4)	97.9 (98.8)	96.4 (96.8)	0.537 (0.506)	−
TargetS	48.4 (50.1)	98.2 (98.3)	96.2 (96.5)	0.492 (0.502)	−

Note: values in brackets are the prediction results of independent testing; SXGBsite is data obtained from Reference (Zhao et al., 2019); EC-RUS, is data obtained from Reference (Ding et al., 2017); TargetS is data obtained from Reference (Yu et al., 2013); the three hyperparameters are the number of convolution layers, filters and batch size, respectively.

**TABLE 4:** Comparison of prediction performance on ATP-388 dataset.

Method	S_n_(%)	S_p_(%)	ACC(%)	MCC	Hyperparameter
S-DCNN	58.97 (50.95)	98.55 (98.99)	97.04 (96.78)	0.5887 (0.5850)	3,32,16
S-SITEatp	69.88 (67.51)	94.47 (92.65)	93.53 (91.51)	0.4550 (0.4160)	−
NsitePred	(46.74)	(97.70)	(95.39)	(0.4560)	−
TargetATPsit	(41.25)	(99.49)	(96.84)	(0.5590)	−
ATPbinding	59.00 (49.40)	98.80 (99.50)	97.30 (97.20)	0.6130 (0.6260)	−
DeepATPseq	52.20 (57.42)	99.03 (99.22)	97.39 (97.32)	0.6130 (0.6550)	−

Note: values in brackets are the prediction results of independent testing.; S-SITEatp, NsitePred and TargetATPsit, are data obtained from Reference (Hu et al., 2018); ATPbinding is data obtained from Reference (Song et al., 2020a); DeepATPseq, is data obtained from Reference (Hu et al., 2021); the three hyperparameters are the number of convolution layers, filters and batch size, respectively.

### Prediction results of independent testing

**FIGURE 7:**
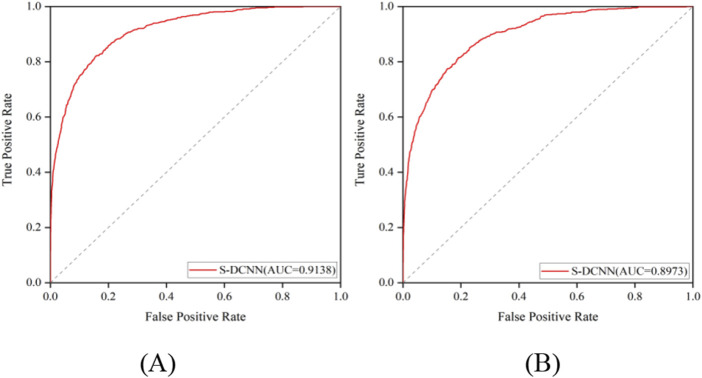
ROC curve of S-DCNN method on ATP-50 **(A)** and ATP-41 set **(B)**.

The prediction results of independent testing were listed in Tables 3, 4, with values displayed in brackets. Notably, Table 3 showcased the enhanced prediction performance of the S-DCNN method. Specifically, on the independent testing set ATP-50, S-DCNN achieved an ACC value of 97.0%, surpassing other methods by 0.2%–0.5%. Concurrently, the S_n_ and MCC values of S-DCNN exhibited notable enhancements, reaching 50.2% and 0.573, respectively, marking a 0.1%–6.5% increase and 3.9%–8.2% improvement compared to alternative methods. The S_p_ value of the S-DCNN was slightly higher than that of other methods. In Table 4, the ACC and MCC values of the S-DCNN on the independent testing set ATP-41 reached 96.78% and 0.5850 respectively. In addition, we drew the ROC curve of the S-DCNN method on the ATP-50 and ATP-41 sets, as shown in Figure 7. The AUC values of the S-DCNN method on the ATP-50 and ATP-41 sets were 0.9138 and 0.8973 respectively.

## Discussion

The comparative analysis in Table 1 revealed that introducing the feature parameters PP, E, and F simultaneously resulted in all four evaluation metrics reaching their maximum values. When compared to the prediction results using parameter B, there were notable increases in the values of S_n_, S_p_, ACC, and MCC. This suggests that incorporating dihedral angles, energy, and propensity factors is beneficial for accurately predicting ATP binding residues. Furthermore, after optimizing the hyperparameters, the prediction results were notably enhanced, with increases of 7.92% in S_n_, 0.39% in S_p_, 0.66% in ACC, and 9.08% in MCC. These results demonstrate the significant performance improvement achieved by optimizing the hyperparameters of the DCNN algorithm.

The data presented in Table 2 highlights that S-DCNN yielded superior prediction results for the evaluation metrics ACC and MCC. Notably, the S_n_ and MCC values of S-DCNN exhibited significant increases of 29.07% and 3.24%, respectively, in comparison to the DCNN outcomes. Similarly, compared to the prediction outcomes of RUS-DCNN, S-DCNN demonstrated substantial enhancements in S_p_, ACC, and MCC values by 24.16%, 20.22%, and 3.24%, respectively. This underscores the efficacy of employing SMOTE-based DCNN in improving prediction performance. Furthermore, in 5-fold cross-validation, S-DCNN demonstrated superior performance over S-SVM with increments in S_n_, S_p_, ACC, and MCC by 6.13%, 1.75%, 1.91%, and 15.1%, respectively, and over S-RF with increases in S_n_ and MCC by 17.06% and 5.41%, respectively. For independent testing, S-DCNN displayed better performances than S-RF, with increases in S_n_ and MCC by 4.67% and 0.81%, and compared to S-SVM with enhancements in S_n_, S_p_, ACC, and MCC by 4.95%, 0.69%, 0.83%, and 9.34%, respectively. Notably, the comparison of AUC values highlighted S-DCNN as the highest predictor, affirming its robustness in identifying ATP binding residues.

The prediction results from the 5-fold cross-validation and independent testing were shown in Tables 3, 4. Compared to SXGBsite (Zhao et al., 2019), EC-RUS (Ding et al., 2017), and TargetS (Yu et al., 2013), S-DCNN showcased improvements in ACC values by 0.6%, 0.6%, and 0.8%, respectively. Notably, when compared with S-SITEatp (Hu et al., 2018), the S_p_, ACC, and MCC values of S-DCNN increased significantly by 4.08%, 3.51%, and 13.37%, respectively. Moreover, the S_n_ value of S-DCNN closely resembled that of ATPbinding (Song et al., 2020b) and surpassed that of DeepATPseq (Hu et al., 2021) by 6.77%. The distinguishing results in independent testing, as detailed in Tables 3, 4 (values in brackets), indicated enhancements in ACC values compared to SXGBsite, EC-RUS, and TargetS by 0.5%, 0.2%, and 0.5%, respectively. Through the analysis in Table 4, S-DCNN presented superiority over S-SITEatp, NsitePred, and TargetATPsit (Hu et al., 2018) in terms of the evaluation metric MCC. Furthermore, it outperformed NsitePred, TargetATPsit, and ATPbinding in the evaluation of S_n_. The performance of S-DCNN closely rivaled that of DeepATPseq. Moreover, the enhanced prediction performance of S-DCNN across diverse datasets was evident from Figures 7A, B, highlighting its robustness. In summary, the S-DCNN method demonstrated consistent reliability.

## Conclusion

To precisely predict ATP binding residues is a critical content for understanding protein function. In this paper, we proposed a novel method of S-DCNN for the prediction of ATP binding residues. Utilizing sequence information, we conducted statistical analysis on dihedral angles, energy, and propensity factors to extract new feature parameters. By optimizing hyperparameters in the S-DCNN predictor, we achieved significantly improved prediction results. Our approach in the S-DCNN involved different data optimization methods. The SMOTE algorithm was employed to prevent information loss in non-binding residue samples, while the DCNN algorithm captured in-depth representation from complex feature parameters with enhanced fault tolerance. Comparative analysis of the prediction results among the DCNN, SVM, and RF algorithms based on SMOTE demonstrated the superiority of the S-DCNN algorithm. Furthermore, applying the S-DCNN predictor to two additional datasets yielded further enhancements in ATP binding residue prediction. In conclusion, the S-DCNN predictor stands out as a robust tool for accurate ATP binding residue prediction. In next step, we will further improve prediction accuracy and build a web server with a user-friendly interface to predict the ATP binding residues.

## Data Availability

The original contributions presented in the study are included in the article/supplementary material, further inquiries can be directed to the corresponding authors.
